# Comparison of two different optical coherence tomography angiography devices in detecting healthy versus glaucomatous eyes – an observational cross-sectional study

**DOI:** 10.1186/s12886-020-01701-9

**Published:** 2020-11-10

**Authors:** A. R. Kee, V. C. H. Yip, E. L. T. Tay, C. W. Lim, J. Cheng, H. Y. Teo, C. H. Chua, L. W. L. Yip

**Affiliations:** 1 National Healthcare Group Eye Institute, Tan Tock Seng Hospital, Singapore, Singapore; 2 Department of Ophthalmology, Woodlands Health Campus, Singapore, Singapore; 3 Department of Ophthalmology, Sarawak General Hospital, Kuching, Malaysia; 4 Cixi Institute of Biomedical Engineering, Chinese Academy of Sciences, Ningbo, Zhejiang Province China; 5 Lee Kong Chian School of Medicine, Nanyang Technological University, Singapore, Singapore

**Keywords:** Optical coherence tomography angiography, AngioVue, Swept source, Glaucoma, Diagnostic, Vascular parameters, Vessel density, Reliability

## Abstract

**Background:**

To understand the differences between two different optical coherence tomography angiography (OCTA) devices in detecting glaucomatous from healthy eyes by comparing their vascular parameters, diagnostic accuracy and test-retest reliability.

**Methods:**

A cross-sectional observational study was performed on healthy and glaucoma subjects, on whom two sets of OCTA images of optic disc and macula were acquired using both AngioVue (Optovue, USA) and Swept Source (Topcon, Japan) OCTA devices during one visit. A novel in-house software was used to calculate the vessel densities. Diagnostic accuracy of the machines in differentiating healthy versus glaucomatous eyes was determined using area under the receiver operating characteristic curve (AUROC) and test-retest repeatability of the machines was also evaluated.

**Results:**

A total of 80 healthy and 38 glaucomatous eyes were evaluated. Glaucomatous eyes had reduced mean vessel density compared to healthy controls in all segmented layers of the optic disc and macula using AngioVue (*p* ≤ 0.001). However, glaucomatous eyes had higher mean vessel density on optic disc scans using Swept Source, with lack of statistically significant difference between healthy and glaucomatous eyes. The AUROC showed better diagnostic accuracy of AngioVue (0.761–1.000) compared to Swept Source (0.113–0.644). The test-retest reliability indices were generally better using AngioVue than Swept Source.

**Conclusions:**

AngioVue showed better diagnostic capability and test-retest reliability compared to Swept Source. Further studies need to be undertaken to evaluate if there is any significant difference between the various machines in diagnosing and monitoring glaucoma.

## Background

Optical coherence tomography angiography (OCTA) is a relatively new, non-invasive imaging modality that is becoming of increasing interest in glaucoma diagnostics. It allows assessment of depth-resolved vascular status by detecting motion contrast from red blood cells and serves as a quick, reproducible, and objective way to qualitatively and quantitatively show areas of altered perfusion in the eye [1, 2]. As vascular dysfunction has been proposed as an etiology for glaucoma [3–6], where loss of retinal vessel density (either as a primary or secondary effect) has been linked to glaucoma development and progression, analysis of ocular blood flow can potentially be used as a means in diagnosing and monitoring patients with glaucoma. Several recent studies have demonstrated microvascular changes using OCTA in optic nerve head, peripapillary area and macular area in glaucomatous eyes [7–12]. In our own recent study, we have found that vessel density at both the disc and macular regions was significantly reduced in glaucomatous eyes compared to controls, and OCTA can demonstrate high diagnostic accuracy in discriminating between glaucoma and healthy subjects using vessel density [13].

There are many OCTA devices available and each manufacturer uses varying techniques to differentiate blood vessels by depicting change in OCT-signal induced by the moving red blood cells. These include but are not limited to: (1) Angiovue OCTA (Optovue RTVue XR Avanti, Optovue Inc., Fremont, CA, USA), which works based on split-spectrum amplitude decorrelation; (2) Swept Source OCT (DRI-OCT Atlantis OCT, Topcon Corporation, Japan), which works based on OCTA ratio analyses algorithm; (3) Zeiss AngioPlex (Cirrus HD-OCT 5000,Zeiss Meditec. Inc.), which works based on micro-angiography; (4) Spectralis OCTA (Heidelberg Engineering, Germany), which works based on full-spectrum amplitude decorrelation algorithm; (5) Prototype of AngioScan (RS-3000 Advance OCT, Nidek Co., Ltd., Japan), which works based on a complex-décor relation algorithm [1, 14, 15].

Due to their technical differences, conversion of parameters and measured values between different instrument systems is difficult. This is perhaps the reason why there is paucity of literature on the comparison between different OCTA instruments in the analysis of vessel density.

In our study, we used 2 different OCTA machines, namely AngioVue and Swept Source OCTA, which are available in our institution, to study the vessel densities in glaucomatous and healthy eyes. We aim to (1) quantitatively evaluate and compare vascular parameters provided by two OCTA devices in glaucomatous and healthy eyes, (2) compare the diagnostic accuracy of vascular parameters in discriminating between glaucomatous and healthy eyes, and (3) compare the test-retest variability within patients.

## Methods

### Study design and patient recruitment

Patients with glaucoma and healthy patients without glaucoma (control group) were recruited from the ophthalmology clinics at Tan Tock Seng Hospital, Singapore, between April 2015 and April 2016. This study is an expansion of patients recruited from our previous studies [13, 16] which evaluated the microvascular density of optic nerve head and macula in healthy and glaucoma subjects using 1 OCTA device. This cross-sectional, prospective, observational study was approved by the Institutional Review Board of the National Healthcare Group and was conducted in accordance to the ethical standards stated in the Declaration of Helsinki. Written informed consent for study participation was obtained from all subjects.

All eligible participants underwent a complete comprehensive ophthalmic examination and investigations, including visual acuity, intraocular pressure (measured via Goldmann applanation tonometry), slit lamp biomicroscopy and fundoscopy. All patients underwent standard automated perimetry (SAP) with Humphrey Field Analyzer Swedish Interactive Threshold Algorithm standard 24–2 (Carl Zeiss Meditec, Dublin, CA). All participants underwent imaging via two different OCTA machines over the peripapillary and macular region on the same day and by the same operator. Vascular parameters of each scan images, as well as the diagnostic accuracy and test-retest reliability of each machine were determined.

Diagnosis of glaucoma was made based on presence of characteristic glaucomatous optic disc changes, such as thinning or notching of optic disc rim and hemorrhages at neuroretinal rim, with no history of other ocular or systemic diseases causing optic nerve damage, and corresponding visual field defects shown via at least two reliable and consistently abnormal SAP. Healthy subjects, who were recruited as controls, had best corrected visual acuity of 6/12 or better, intraocular pressure lower than 22 mmHg, normal-appearing optic disc and retina, and normal SAP.

The following exclusion criteria were used: (1) history of intraocular surgery or previous laser therapy (but history of laser or surgery performed for the treatment of glaucoma allowed in the glaucoma group) (2) co-existing ocular pathologies such as vascular or non-vascular retinopathies, non-glaucomatous optic neuropathy (3) co-existing ocular or systemic diseases known to produce visual field defect (4) subjects known to be pregnant at time of recruitment (5) subjects younger than 21 years of age. In cases where both eyes of a normal or glaucomatous patient were eligible, both eyes from each patient were included in the study.

### Optical coherence tomography angiography

#### Image acquisition

The AngioVue Enhanced Microvascular Imaging System (Optovue, Inc., Fremont, CA, USA) uses an 840 nm wavelength diode laser source and has an A-scan rate of 70,000 scans per second [17]. Each OCTA volume consists of 304 × 304 A-scan with 2 consecutive B-scans captured at each fixed position before proceeding to the next sampling location. The split-spectrum amplitude-decorrelation angiography (SSADA) algorithm was used to capture motion-contrast blood flow images and provide high-resolution 3-dimentional visualisation of perfused posterior segment microvasculature.

The Swept Source OCTA (SS-OCTA) (DRI-OCT Atlantis OCT, Topcon Corporation, Japan) uses a 1050 nm wavelength system and has an A-scan rate of 100,000 scans per second. Each B-scan position was repeatedly scanned 4 times for OCTA processing using an evaluation version of the OCTA software. Swept Source OCTA uses a ratio method, named OCTA Ratio Analysis (OCTARA), in which the full spectrum is kept intact, as compared to SSADA, and hence allowing preservation of axial resolution. The principle of ratio method has been well explained by Stanga et al. [18].

We followed the manufacturers’ recommended scanning technique to capture images. Subjects were positioned with good eye alignment, and steady gaze was maintained via the systems’ internal fixation target. The two machines’ auto-focus technology was used to provide accurate focus on the posterior segment structure (optic disc or macula) of interest. Subjects were advised to maintain stable head position and gaze during scanning, but allowed to rest, blink or reposition in between scans.

Each subjects had up to two images taken per peripapillary and macular region for assessment of repeatability. Imaging of optic nerve head was performed with 3.0 × 3.0 mm scans centred on optic nerve head for both AngioVue and Swept Source OCTA machines whereas imaging of macula was obtained using 3.0 × 3.0 mm and 6.0 × 6.0 mm scans centred on fovea macular scan for AngioVue and Swept Source OCTA devices respectively. After each scanning process, the images were reviewed and filtered. Poor quality images, i.e. images with significant motion artifacts as evidenced by irregular vessel pattern or disc boundary on the en face angiogram, poor image clarity or poor signal strength (signal strength index < 40), were excluded from the study.

#### Segmentation and subdivision

**Fig. 1:**
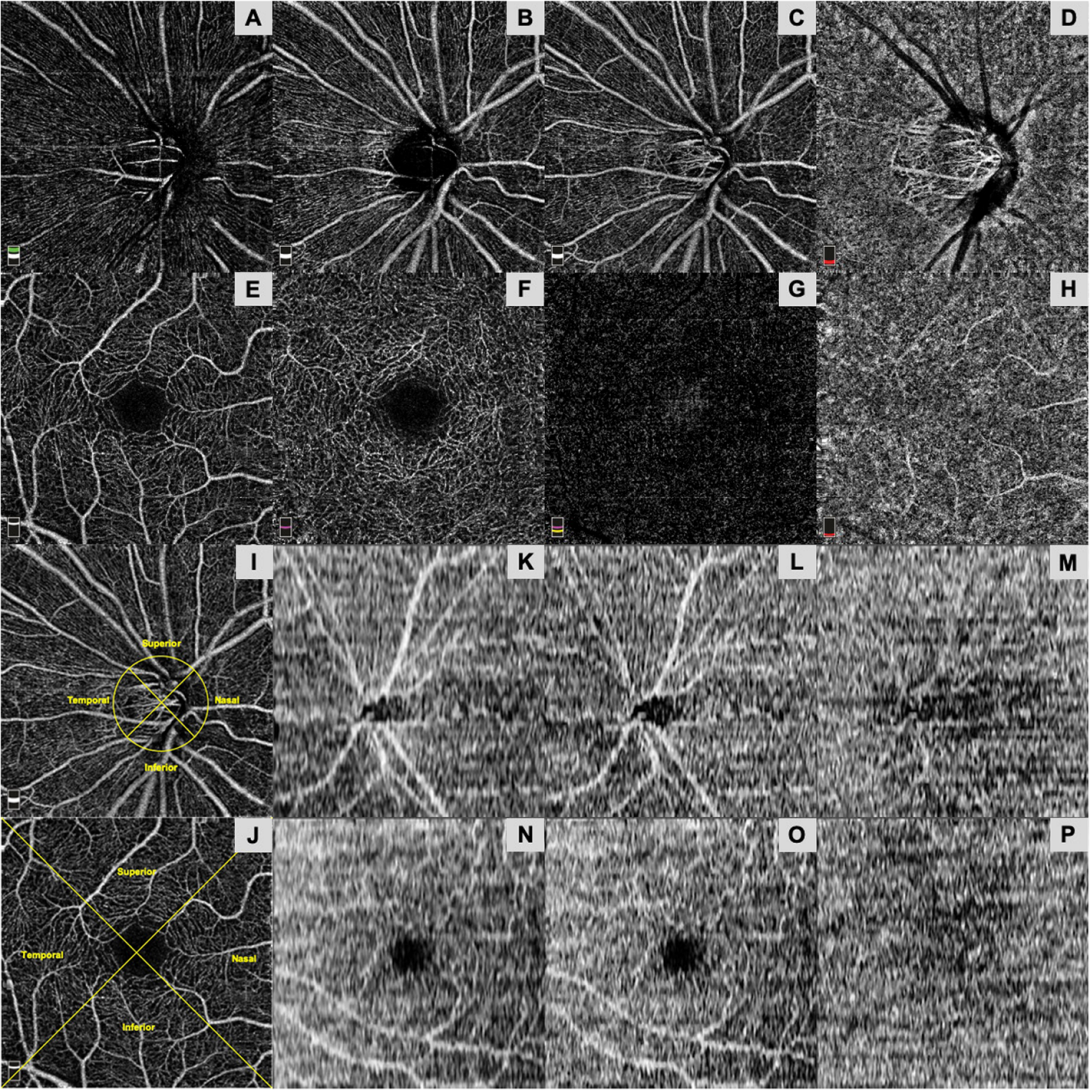
Representative images from AngioVue and SweptSource optical coherence tomography angiography (OCTA) devices. Optic disc scans using AngioVue are divided into (**a**) vitreous, (**b**) radial peripapillary capillaries, (**c**) nerve head, and (**d**) disc choroid layers. Macular scans using AngioVue are divided into (**e**) superficial retina, (**f**) deep retina, (**g**) outer retina, and (**h**) choroid layers. Example of quadrant division of (**i**) optic disc scan and (**j**) macular scan using AngioVue. Optic disc scans (raw images shown) using SweptSource are divided into (**k**) superficial retina, (**l**) deep retina, and (**m**) choroid layers. Similarly, macular scans (raw images shown) using SweptSource are divided into (**n**) superficial retina, (**o**) deep retina, and (**p**) choroid layers

Both AngioVue and Swept Source softwares were able to perform automated layer segmentation of the optic disc and macular regions into specific areas or layers (Fig. 1). AngioVue segments optic disc scan (vitreous, radial peripapillary capillaries (RPC), nerve head, disc choroid) and macular scan (superficial retina, deep retina, outer retina, choroid) into 4 layers whereas Swept Source segments both optic disc and macular scans into 3 layers (superficial retina, deep retina, choroid)**.**

This generated detailed en face images, allowing visualisation of vascular details within these segmented boundaries. Each en face image layer was thereafter further subdivided into 4 equal quadrants, namely superior, inferior, temporal and nasal quadrant (Fig. 1). The four quadrants are determined based on a diagonal and anti-diagonal line.

#### Vessel density calculation

Vessel density of each en face layer and quadrant of optic disc and macula was analysed using a novel in-house developed software involving vessel detection and pixel counting. This is described in detail in our recent study [13].

### Statistical analysis

Statistical analysis was performed using IBM SPSS Statistics version 22 (IBM Corp, New York, USA). Statistical significance was defined by *p*-value less than 0.05. Descriptive statistics included mean and standard deviation for normally distributed variables.

To account the use of both eyes (right and left eye) from the same patient, we used linear mix model to compare between healthy and glaucomatous eyes while controlling for age and gender. In linear mixed model, the vessel density is treated as dependent variable, whereas, age, gender and grouping [glaucomatous vs healthy (control)] are fixed factors. The observation for each patient’s right and left eye was treated as repeated observation, with repeated covariance type set as: Compound symmetric.

Diagnostic accuracy of both OCTA devices for differentiating between healthy and glaucomatous eyes was evaluated by calculating the Area Under the Receiver Operating Characteristic (AUROC) curve. AUROC analyses were performed in Stata software Version 16 (StataCorp, College Station, Texas, USA) with the ROC regression method proposed by Alonzo and Pepe [19] and Janes et al. [20]. We employed a non-parametric ROC regression analysis (*rocreg*) with linear covariate adjustment (by specifying *ctrlmodel (linear)*) for patient age and gender, and cluster adjustment (by specifying *cluster*) for patient tested in each eye (right and left eye).

Repeatability was assessed by within-subject standard deviation (Sw) and within-subject coefficient of variation (CV). The Sw was calculated as the square root of (di2)/2n where di is the difference between measurement for each individual and n is the number of subjects. The CV (100 x Sw / overall mean) was calculated according to the procedure described by Bland and Altman.

## Results

### Study population

82 healthy control eyes and 39 glaucomatous eyes were initially included. Of these eyes, 3 were excluded due to poor quality OCTA images. A total of 80 control eyes and 38 glaucomatous eyes were studied.

Mean age of participants was 44.8 +/− 12.8 and 64.3 +/− 6.28 in the control and glaucoma group respectively (*p* < 0.001). Given that age can be a factor in affecting vessel densities, we adjusted for age in all our statistical analysis, including comparisons between healthy and glaucomatous eyes and AUROC. Baseline characteristics of both control and glaucoma patients are listed in Table 1.

**Table 1:** Baseline characteristics of control and glaucoma group

		Control (40 patients, *n* = 80 eyes)	Glaucoma (30 patients, *n* = 38 eyes)	***P***-value
**Age (mean year +/− SD)**		44.8 ± 12.8	64.3 ± 6.28	< 0.001
**Gender** [n (%)]	Male	17 (42.5%)	26 (86.7%)	< 0.001
	Female	23 (57.5%)	4 (13.3%)	
**Ethnicity** [n (%)]	Chinese	34 (85.0%)	26 (86.7%)	0.773
	Malay	2 (5.0%)	1 (3.3%)	
	Indian	3 (7.5%)	1 (3.3%)	
	Others	1 (2.5%)	2 (6.7%)	
**Presence of cataract** [n (%)]	Yes	6 (15.0%)	24 (80.0%)	< 0.001
	No	34 (85.0%)	6 (20.0%)	
**Intraocular pressure** (mean mmHg +/− SD)		13.64 ± 2.72	14.89 ± 2.38	0.018
**HVF** (mean MD +/− SD)		–	−8.51 ± 6.98	–
**HVF** (mean PSD +/− SD)		–	7.46 ± 3.93	–
**HVF** (mean VFI +/− SD)		–	80.53 ± 17.17	–
**Type of glaucoma** [n (%)]	Open angle	–	33 (86.8%)	–
	Closed angle	–	5 (13.2%)	–

*SD* Standard deviation; *HVF* Humphrey visual field; *MD* Mean deviation; *PSD* Pattern standard deviation; *VFI* Visual field index

### Vessel density

Statistically significant differences were found between healthy and glaucomatous eyes for almost all vessel density parameters using AngioVue (Tables 2, 3). For glaucoma group, the age and gender-adjusted mean vessel density of optic nerve head at the choroid, nerve head, RPCs and vitreous was 0.177 ± 0.016, 0.084 ± 0.009, 0.042 ± 0.009, 0.052 ± 0.010 (Table 2) whereas that of macula at the level of choroid, outer retina, deep retina, and superficial retina was 0.133 ± 0.011, 0.089 ± 0.013, 0.117 ± 0.008 and 0.076 ± 0.004 respectively (Table 3). For both optic disc and macula scans, the mean vessel density of each segmented layer was reduced in glaucoma patients compared to healthy controls (*p* ≤ 0.001).

**Table 2:** Comparison of vessel density of optic disc scans between control and glaucoma group using AngioVue

Layers	Age and gender-adjusted vessel density (%)				***P***-value
	Control (***n*** = 80)		Glaucoma (n = 38)		
	Mean ± Standard Error	95% Confidence Interval	Mean ± Standard Error	95% Confidence Interval	
**Vitreous**					
Inferior	0.120 ± 0.009	0.101–0.138	0.099 ± 0.017	0.066–0.133	0.352
Superior	0.094 ± 0.010	0.074–0.114	0.056 ± 0.016	0.024–0.089	0.090
Nasal	0.099 ± 0.010	0.079–0.118	0.062 ± 0.015	0.032–0.092	0.078
Temporal	0.100 ± 0.008	0.084–0.116	0.073 ± 0.013	0.047–0.099	0.123
Mean	0.100 ± 0.006	0.087–0.112	0.052 ± 0.010	0.032–0.073	0.001
**Radial peripapillary capillaries**					
Inferior	0.188 ± 0.009	0.170–0.205	0.069 ± 0.015	0.039–0.099	< 0.001
Superior	0.128 ± 0.010	0.108–0.147	0.040 ± 0.016	0.008–0.073	< 0.001
Nasal	0.155 ± 0.011	0.133–0.176	0.058 ± 0.018	0.021–0.094	< 0.001
Temporal	0.087 ± 0.008	0.071–0.104	0.049 ± 0.016	0.018–0.081	0.061
Mean	0.139 ± 0.006	0.128–0.150	0.042 ± 0.009	0.023–0.060	< 0.001
**Nerve head**					
Inferior	0.220 ± 0.009	0.202–0.237	0.117 ± 0.015	0.088–0.147	< 0.001
Superior	0.211 ± 0.009	0.193–0.229	0.069 ± 0.015	0.039–0.099	< 0.001
Nasal	0.219 ± 0.008	0.202–0.235	0.089 ± 0.014	0.061–0.117	< 0.001
Temporal	0.154 ± 0.010	0.135–0.173	0.065 ± 0.016	0.034–0.097	< 0.001
Mean	0.200 ± 0.005	0.190–0.211	0.084 ± 0.009	0.066–0.101	< 0.001
**Choroid**					
Inferior	0.243 ± 0.011	0.222–0.265	0.199 ± 0.018	0.164–0.234	0.060
Superior	0.303 ± 0.013	0.276–0.330	0.179 ± 0.012	0.137–0.222	< 0.001
Nasal	0.256 ± 0.012	0.233–0.280	0.184 ± 0.020	0.146–0.223	0.007
Temporal	0.227 ± 0.013	0.200–0.253	0.144 ± 0.022	0.101–0.187	0.005
Mean	0.256 ± 0.010	0.236–0.276	0.177 ± 0.016	0.145–0.209	0.001

**Table 3:** Comparison of vessel density of macula scans between control and glaucoma group using AngioVue

Layers	Age and gender -adjusted vessel density (%)				***P***-value
	Control (***n*** = 80)		Glaucoma (***n*** = 38)		
	Mean ± Standard Error	95% Confidence Interval	Mean ± Standard Error	95% Confidence Interval	
**Superficial retina**					
Inferior	0.126 ± 0.004	0.118–0.133	0.082 ± 0.005	0.071–0.093	< 0.001
Superior	0.122 ± 0.003	0.115–0.128	0.086 ± 0.005	0.077–0.096	< 0.001
Nasal	0.125 ± 0.010	0.104–0.146	0.067 ± 0.015	0.037–0.098	0.011
Temporal	0.127 ± 0.006	0.115–0.139	0.058 ± 0.009	0.040–0.075	< 0.001
Mean	0.116 ± 0.002	0.111–0.121	0.076 ± 0.004	0.069–0.084	< 0.001
**Deep retina**					
Inferior	0.239 ± 0.007	0.225–0.252	0.118 ± 0.010	0.098–0.137	< 0.001
Superior	0.233 ± 0.005	0.222–0.243	0.108 ± 0.008	0.093–0.124	< 0.001
Nasal	0.250 ± 0.008	0.235–0.265	0.115 ± 0.011	0.092–0.138	< 0.001
Temporal	0.265 ± 0.011	0.243–0.287	0.114 ± 0.016	0.082–0.146	< 0.001
Mean	0.244 ± 0.006	0.232–0.255	0.117 ± 0.008	0.100–0.133	< 0.001
**Outer retina**					
Inferior	0.159 ± 0.012	0.135–0.182	0.091 ± 0.017	0.057–0.125	0.008
Superior	0.146 ± 0.014	0.118–0.173	0.072 ± 0.019	0.034–0.110	0.010
Nasal	0.207 ± 0.015	0.177–0.237	0.089 ± 0.023	0.043–0.134	0.001
Temporal	0.198 ± 0.015	0.166–0.229	0.118 ± 0.022	0.074–0.163	0.017
Mean	0.186 ± 0.009	0.167–0.205	0.089 ± 0.013	0.062–0.115	< 0.001
**Choroid**					
Inferior	0.251 ± 0.008	0.236–0.267	0.128 ± 0.011	0.105–0.150	< 0.001
Superior	0.245 ± 0.009	0.227–0.264	0.134 ± 0.013	0.108–0.160	< 0.001
Nasal	0.240 ± 0.013	0.213–0.266	0.139 ± 0.019	0.102–0.177	< 0.001
Temporal	0.253 ± 0.009	0.234–0.272	0.133 ± 0.014	0.106–0.160	< 0.001
Mean	0.251 ± 0.008	0.235–0.266	0.133 ± 0.011	0.111–0.154	< 0.001

On the other hand, more variable results were found for OCTA parameters using Swept Source (Tables 4, 5). For glaucoma group, the mean vessel density of optic nerve head at the choroid, deep retina and superficial retina was 0.440 ± 0.024, 0.295 ± 0.022, and 0.294 ± 0.026 respectively whereas that of macula at level of choroid, deep retina and superficial retina was 0.319 ± 0.021, 0.236 ± 0.022, and 0.207 ± 0.021 respectively. The difference between healthy and glaucomatous eyes was not statistically significant, except for temporal quadrant (*p* = 0.040) and the mean vessel density (*p* = 0.048) of deep retina segment of optic disc (p = 0.040) as well as inferior quadrant (*p* = 0.001), temporal quadrant (*p* = 0.003) and the mean vessel density (*p* = 0.007) of choroid layer of optic disc. Similar to the findings from the AngioVue, the mean vessel density of the macula scans was generally lower in glaucoma patients compared to healthy controls, except for temporal quadrants of superficial retina and deep retina layers. However, contrary to the findings from AngioVue, the mean vessel density of the optic disc scans was generally higher in glaucoma patients compared to healthy controls, except for inferior quadrant of superficial retinal layer of optic disc.

**Table 4:** Comparison of vessel density of optic disc scans between control and glaucoma group using SweptSource

Layers	Age and gender -adjusted vessel density (%)				***P***-value
	Control (***n*** = 80)		Glaucoma (***n*** = 38)		
	Mean ± Standard Error	95% Confidence Interval	Mean ± Standard Error	95% Confidence Interval	
**Superficial retina**					
Inferior	0.316 ± 0.018	0.281–0.351	0.273 ± 0.030	0.313–0.432	0.156
Superior	0.241 ± 0.019	0.202–0.279	0.270 ± 0.033	0.204–0.335	0.500
Nasal	0.181 ± 0.016	0.149–0.213	0.251 ± 0.031	0.190–0.312	0.074
Temporal	0.203 ± 0.018	0.167–0.240	0.280 ± 0.031	0.217–0.342	0.066
Mean	0.235 ± 0.015	0.204–0.265	0.294 ± 0.026	0.241–0.346	0.089
**Deep retina**					
Inferior	0.266 ± 0.016	0.234–0.298	0.331 ± 0.026	0.279–0.384	0.065
Superior	0.233 ± 0.018	0.196–0.270	0.261 ± 0.031	0.198–0.323	0.504
Nasal	0.242 ± 0.013	0.215–0.269	0.295 ± 0.026	0.244–0.347	0.105
Temporal	0.213 ± 0.017	0.180–0.246	0.292 ± 0.029	0.234–0.351	0.040
Mean	0.238 ± 0.012	0.214–0.263	0.295 ± 0.022	0.252–0.338	0.048
**Choroid**					
Inferior	0.355 ± 0.016	0.322–0.388	0.479 ± 0.027	0.424–0.533	0.001
Superior	0.339 ± 0.019	0.301–0.378	0.394 ± 0.032	0.330–0.457	0.202
Nasal	0.355 ± 0.018	0.319–0.391	0.426 ± 0.031	0.365–0.487	0.079
Temporal	0.351 ± 0.017	0.318–0.385	0.463 ± 0.028	0.408–0.518	0.003
Mean	0.350 ± 0.015	0.321–0.379	0.440 ± 0.024	0.391–0.489	0.007

**Table 5:** Comparison of vessel density of macula scans between control and glaucoma group using SweptSource

Layers	Age and gender -adjusted vessel density (%)				***P***-value
	Control (***n*** = 80)		Glaucoma (***n*** = 38)		
	Mean ± Standard Error	95% Confidence Interval	Mean ± Standard Error	95% Confidence Interval	
**Superficial retina**					
Inferior	0.323 ± 0.017	0.288–0.358	0.255 ± 0.031	0.194–0.315	0.087
Superior	0.188 ± 0.013	0.162–0.214	0.131 ± 0.023	0.086–0.177	0.057
Nasal	0.196 ± 0.015	0.166–0.225	0.181 ± 0.028	0.126–0.236	0.669
Temporal	0.260 ± 0.016	0.228–0.293	0.269 ± 0.031	0.207–0.330	0.831
Mean	0.241 ± 0.011	0.218–0.264	0.207 ± 0.021	0.166–0.248	0.199
**Deep retina**					
Inferior	0.315 ± 0.018	0.280–0.351	0.288 ± 0.032	0.224–0.351	0.500
Superior	0.201 ± 0.017	0.167–0.236	0.176 ± 0.029	0.118–0.234	0.504
Nasal	0.240 ± 0.016	0.208–0.272	0.207 ± 0.029	0.148–0.265	0.375
Temporal	0.263 ± 0.017	0.229–0.297	0.281 ± 0.032	0.218–0.344	0.654
Mean	0.255 ± 0.012	0.230–0.279	0.236 ± 0.022	0.193–0.280	0.521
**Choroid**					
Inferior	0.357 ± 0.015	0.326–0.387	0.328 ± 0.027	0.274–0.382	0.415
Superior	0.317 ± 0.016	0.286–0.348	0.302 ± 0.026	0.250–0.354	0.670
Nasal	0.357 ± 0.015	0.327–0.387	0.353 ± 0.027	0.300–0.407	0.918
Temporal	0.357 ± 0.014	0.329–0.386	0.299 ± 0.026	0.48–0.350	0.082
Mean	0.347 ± 0.012	0.324–0.370	0.319 ± 0.021	0.277–0.360	0.292

**Fig. 2:**
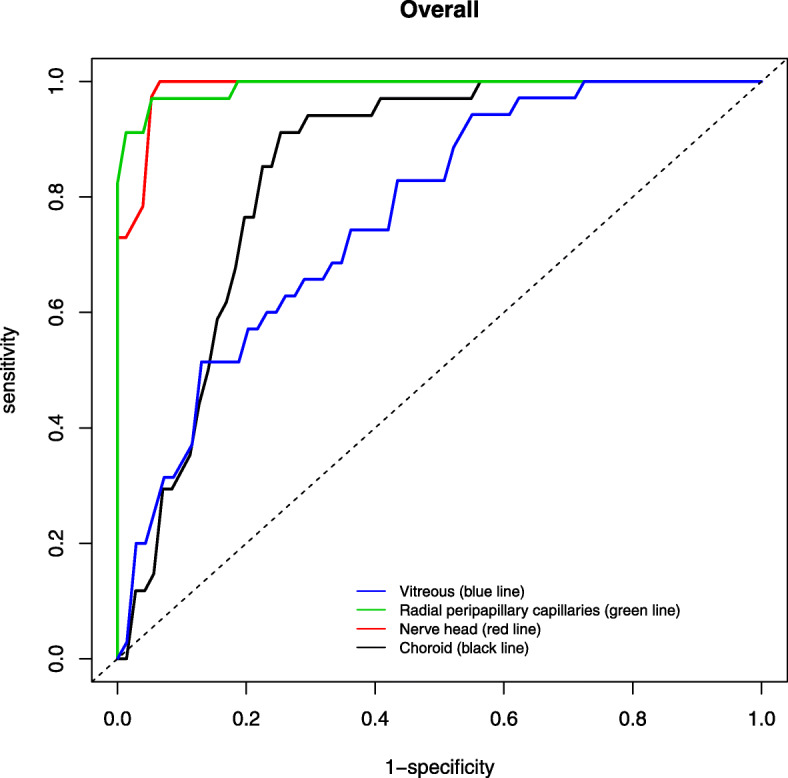
Area under the receiver operating characteristic (AUROC) of optic disc scan using AngioVue

**Fig. 3:**
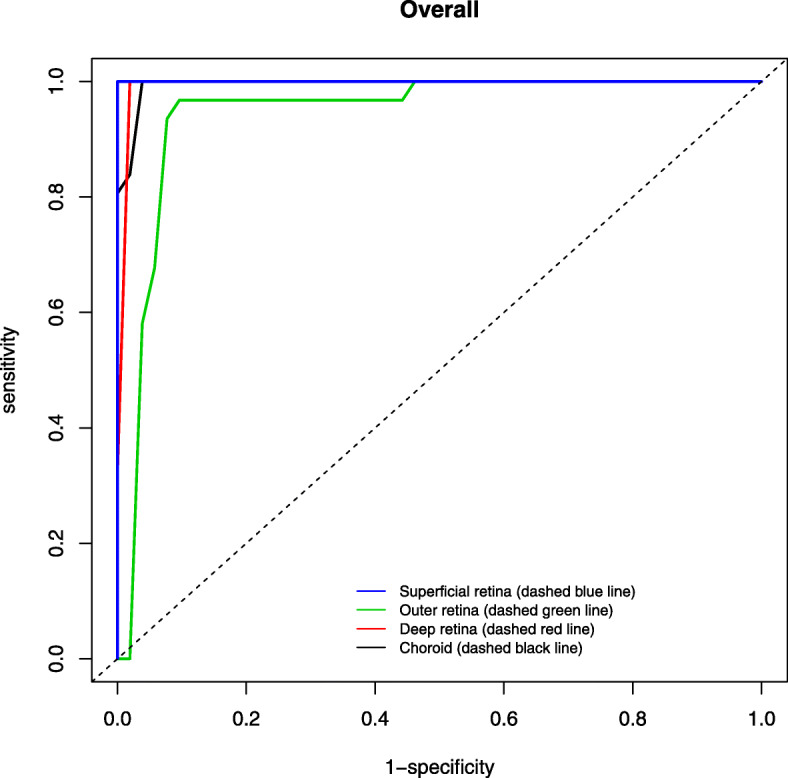
Area under the receiver operating characteristic (AUROC) of macula scan using AngioVue

**Fig. 4:**
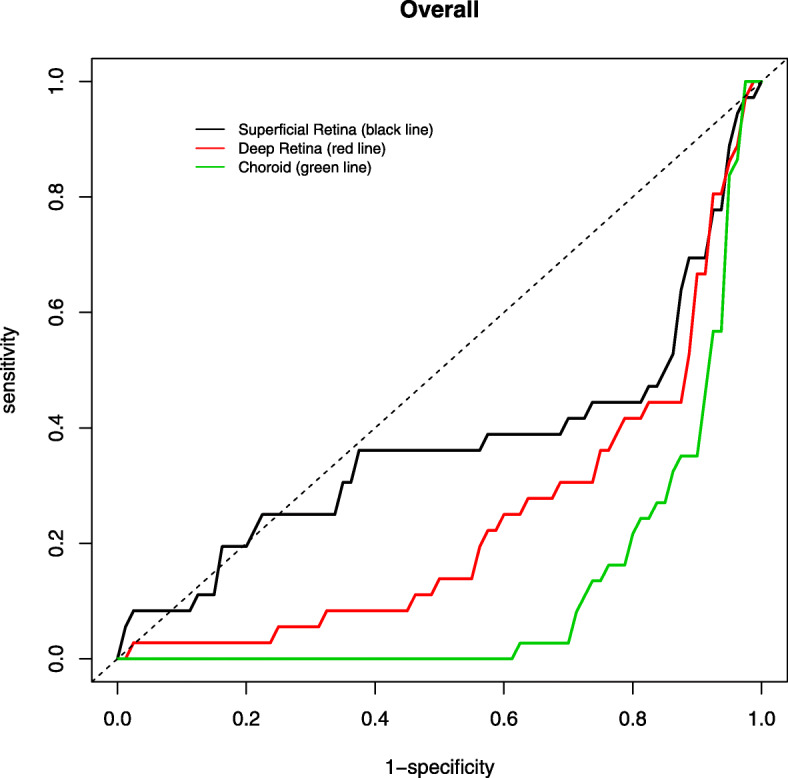
Area under the receiver operating characteristic (AUROC) of optic disc scan using SweptSource

**Fig. 5:**
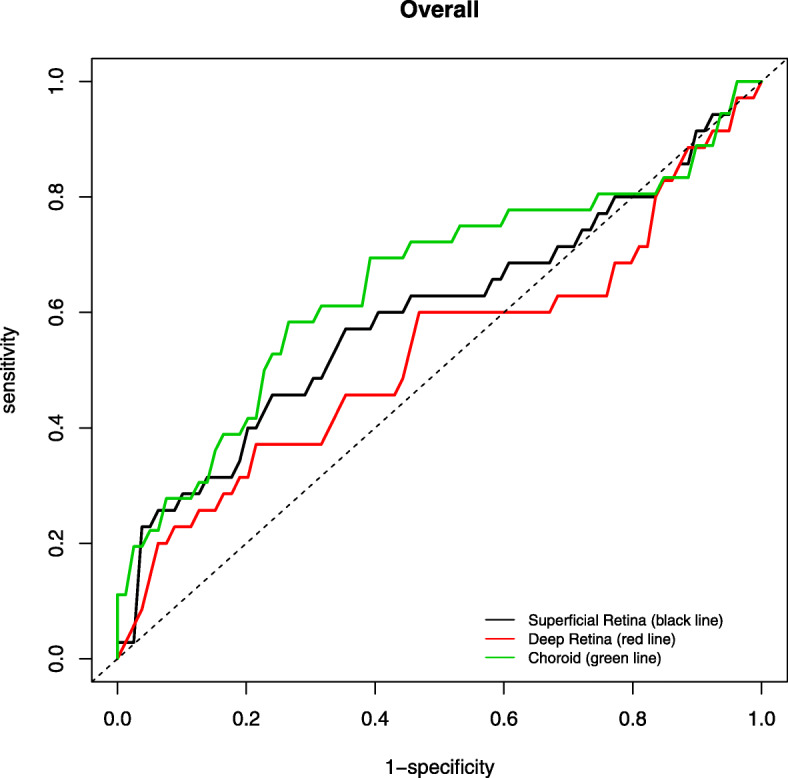
Area under the receiver operating characteristic (AUROC) of macula scan using SweptSource

The AUROC for discriminating between healthy and glaucomatous eyes using AngioVue (Figs. 2, 3) was highest for RPC (0.990) followed by nerve head (0.986), choroid (0.840) and vitreous (0.761) for optic disc whilst highest for superficial retina (1.000), followed by choroid (0.993), deep retina (0.987) and outer retina (0.934) for macular area (Table 6). In contrast, the AUROC for distinguishing between healthy and glaucomatous eyes using Swept Source was generally lower (Figs. 4, 5), with highest being superficial retina (0.365), followed by deep retina (0.232) and choroid (0.113) layers of optic disc and choroid (0.644) followed by superficial retina (0.591) and deep retina (0.525) layers of macula (Table 7).

**Table 6:** Area under the receiver operating characteristic (AUROC) values for optic disc and macula scans in discriminating healthy and glaucomatous eyes using AngioVue

Optical Disc			Macula		
**Layers**	**Age and Gender - adjusted**		**Layers**	**Age and Gender - adjusted**	
	**AUC**	**95% Confidence Interval**		**AUC**	**95% Confidence Interval**
**Vitreous**			**Superficial retina**		
Inferior	0.569	0.424–0.713	Inferior	0.937	0.873–1.000
Superior	0.585	0.394–0.777	Superior	0.954	0.897–1.000
Nasal	0.703	0.475–0.931	Nasal	0.988	0.924–1.000
Temporal	0.692	0.521–0.863	Temporal	0.994	0.977–1.000
Mean	0.761	0.616–0.907	Mean	1.000	0.995–1.000
**Radial peripapillary capillaries**			**Outer retina**		
Inferior	0.920	0.853–0.987	Inferior	0.818	0.592–1.000
Superior	0.830	0.693–0.968	Superior	0.785	0.550–1.000
Nasal	0.907	0.822–0.992	Nasal	0.882	0.743–1.000
Temporal	0.729	0.543–0.915	Temporal	0.855	0.695–1.000
Mean	0.990	0.973–1.000	Mean	0.934	0.825–1.000
**Nerve head**			**Deep retina**		
Inferior	0.864	0.755–0.973	Inferior	0.984	0.953–1.000
Superior	0.947	0.885–1.000	Superior	0.999	0.994–1.000
Nasal	0.931	0.886–0.976	Nasal	0.980	0.940–1.000
Temporal	0.818	0.688–0.948	Temporal	0.960	0.873–1.000
Mean	0.986	0.963–1.000	Mean	0.987	0.961–1.000
**Choroid**			**Choroid**		
Inferior	0.654	0.484–0.825	Inferior	0.985	0.956–1.000
Superior	0.866	0.764–0.968	Superior	0.980	0.910–1.000
Nasal	0.694	0.551–0.836	Nasal	0.837	0.642–0.972
Temporal	0.762	0.608–0.916	Temporal	0.966	0.902–1.000
Mean	0.840	0.705–0.976	Mean	0.993	0.961–1.000

**Table 7:** Area under the receiver operating characteristic (AUROC) values for optic disc and macular scans in discriminating healthy and glaucomatous eyes using SweptSource

Optical Disc			Macula		
**Layers**	**Age and Gender - adjusted**		**Layers**	**Age and Gender - adjusted**	
	**AUC**	**95% Confidence Interval**		**AUC**	**95% Confidence Interval**
**Superficial retina**			**Superficial retina**		
Inferior	0.391	0.211–0.571	Inferior	0.598	0.432–0.764
Superior	0.554	0.249–0.859	Superior	0.694	0.512–0.877
Nasal	0.336	0.069–0.603	Nasal	0.541	0.374–0.709
Temporal	0.294	0.088–0.500	Temporal	0.369	0.210–0.527
Mean	0.365	0.082–0.649	Mean	0.591	0.374–0.809
**Deep retina**			**Deep retina**		
Inferior	0.265	0.103–0.427	Inferior	0.448	0.276–0.621
Superior	0.450	0.069–0.832	Superior	0.643	0.440–0.846
Nasal	0.391	0.174–0.609	Nasal	0.576	0.383–0.770
Temporal	0.239	0.082–0.397	Temporal	0.363	0.208–0.517
Mean	0.232	0.000–0.472	Mean	0.525	0.272–0.779
**Choroid**			**Choroid**		
Inferior	0.170	0.063–0.277	Inferior	0.492	0.320–0.663
Superior	0.370	0.096–0.643	Superior	0.606	0.400–0.813
Nasal	0.281	0.073–0.489	Nasal	0.462	0.250–0.674
Temporal	0.235	0.035–0.436	Temporal	0.660	0.491–0.829
Mean	0.113	0.000–0.311	Mean	0.644	0.406–0.882

**Fig. 6:**
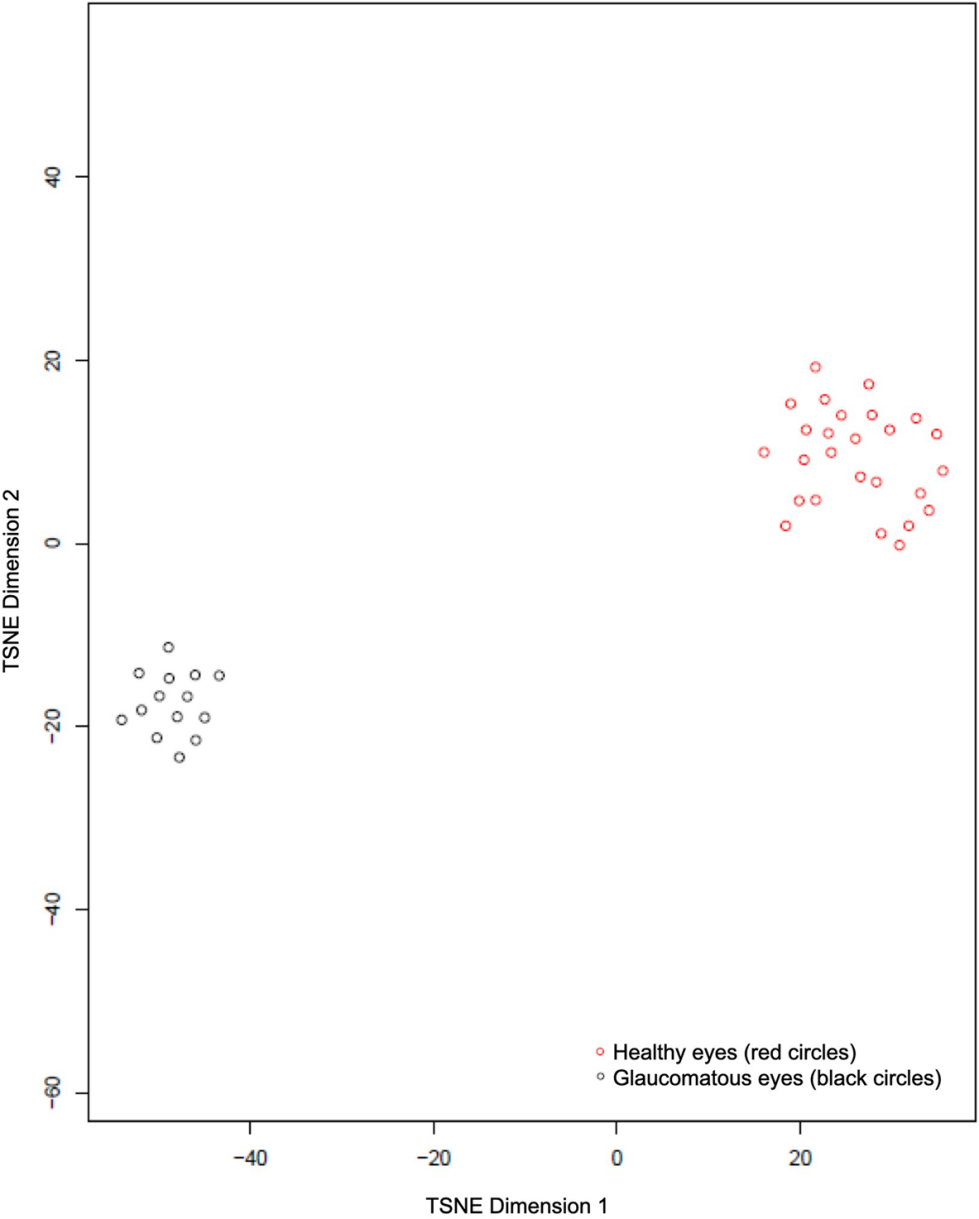
T-distributed Stochastic Neighbour Embedding (tSNE) plot for healthy versus glaucomatous eyes using AngioVue

**Fig. 7:**
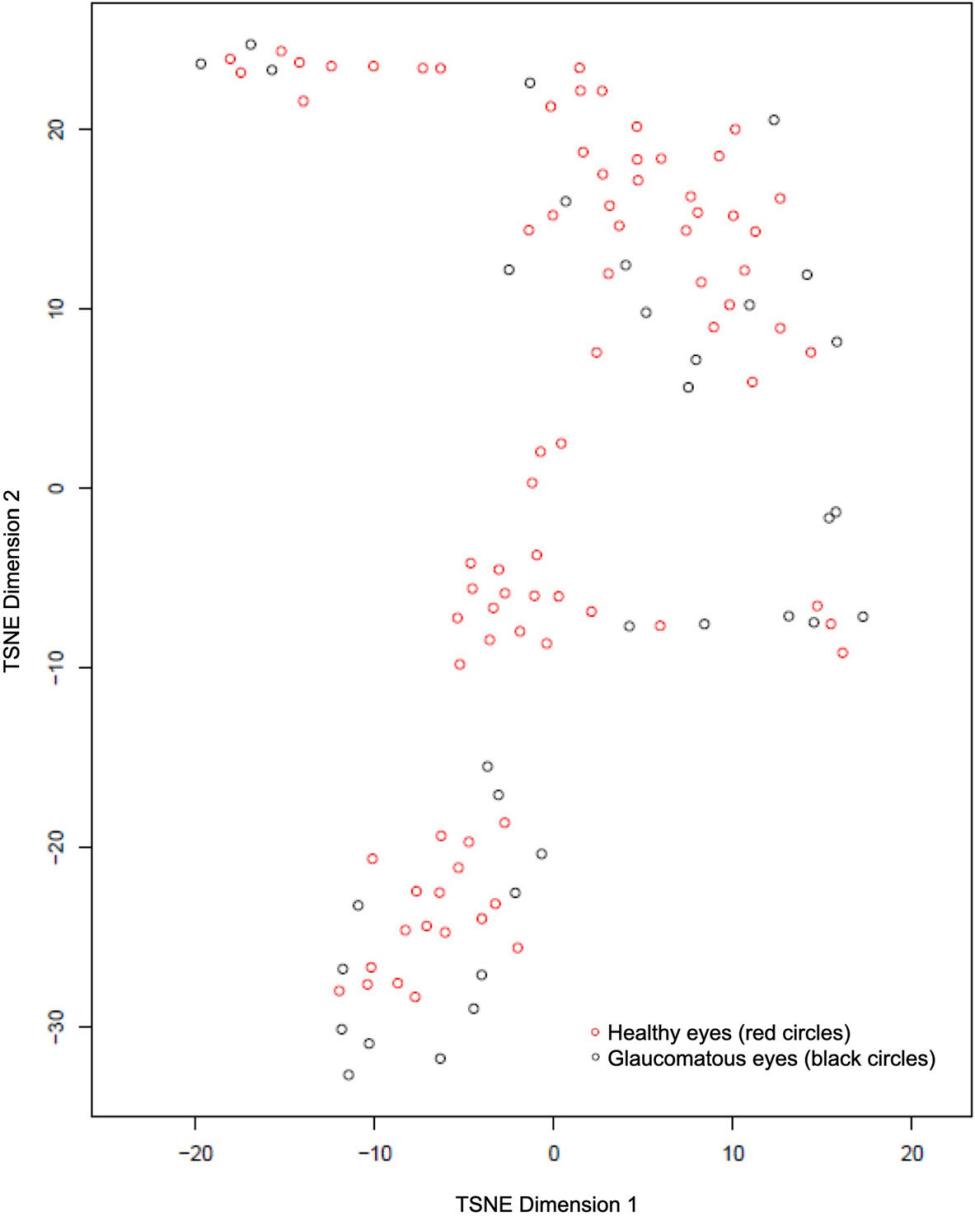
T-distributed Stochastic Neighbour Embedding (tSNE) plot for healthy versus glaucomatous eyes using SweptSource

We used T-distributed Stochastic Neighbour Embedding (tSNE) plots (Figs. 6 and 7), which are based on a technique of dimensionality reduction for visualization of high-dimensional datasets, to show graphical representations of all statistical parameters of healthy and glaucomatous eyes in AngioVue and Swept Source. The tSNE plots show that datasets between healthy and glaucomatous eyes are easily distinguishable using AngioVue but not using Swept Source, which is consistent with our finding that there is a more promising distinguishing ability between glaucoma and control patients using AngioVue than that using Swept Source.

### Test retest variability

For AngioVue, the within-subject standard deviation (Sw) of optic disc scans ranged from 0.022–0.042 for healthy eyes and 0.012–0.027 for glaucomatous eyes whereas coefficient of variation (CV) of optic disc scans ranged from 15 to 26% for healthy eyes and 15–33% in glaucomatous eyes (Table 8). Sw of macular scans ranged from 0.016–0.027 for healthy eyes and 0.007–0.012 for glaucomatous eyes while CV ranged from 7 to 14% for both healthy and glaucomatous eyes.

**Table 8:** Test-retest reliability of optic disc and macular scans of glaucomatous and healthy eyes using AngioVue

Eyes	Scan	Layers	Overall mean	Sw	Sw 95% CI	CV	CR_**w**_	CR_**w**_ 95% CI
Glaucoma	Optic disc	Vitreous	0.054	0.012	0.009–0.015	22	0.033	0.025–0.041
		Radial peripapillary capillaries	0.050	0.016	0.013–0.020	33	0.046	0.035–0.057
		Nerve head	0.095	0.016	0.013–0.020	17	0.045	0.035–0.055
		Choroid	0.178	0.027	0.021–0.033	15	0.075	0.057–0.092
	Macula	Superficial retina	0.074	0.007	0.005–0.008	9	0.018	0.014–0.023
		Deep retina	0.106	0.011	0.008–0.013	10	0.030	0.022–0.037
		Outer retina	0.083	0.012	0.009–0.015	14	0.033	0.025–0.041
		Choroid	0.128	0.010	0.007–0.012	7	0.027	0.020–0.033
Control	Optic disc	Vitreous	0.103	0.027	0.022–0.031	26	0.074	0.062–0.087
		Radial peripapillary capillaries	0.135	0.022	0.019–0.026	17	0.062	0.052–0.072
		Nerve head	0.197	0.029	0.025–0.034	15	0.081	0.068–0.094
		Choroid	0.264	0.042	0.035–0.049	16	0.117	0.098–0.137
	Macula	Superficial retina	0.118	0.016	0.013–0.019	14	0.045	0.036–0.053
		Deep retina	0.251	0.017	0.013–0.020	7	0.046	0.037–0.055
		Outer retina	0.188	0.027	0.022–0.032	14	0.075	0.061–0.089
		Choroid	0.255	0.020	0.016–0.024	8	0.056	0.045–0.066

*Sw* Within-subject standard deviation; *CI* Confidence interval; *CV* Coefficient of variation; *CR*_*w*_ Coefficient of repeatability

For Swept Source, Sw of optic disc scans was 0.065–0.068 and 0.117–0.125 and CV of 20–28% and 29–44% for healthy and glaucomatous eyes respectively (Table 9). Similarly, repeatability was poorer for Swept Source than AngioVue for macular scans, where Sw was 0.106–0.115 and 0.114–0.120 while CV was 32–49% and 29–49% for control group and glaucoma group respectively.

**Table 9:** Test-retest reliability of optic disc and macular scans of glaucomatous and healthy eyes using SweptSource

Eyes	Scan	Layers	Overall mean	Sw	Sw 95% CI	CV	CR_**w**_	CR_**w**_ 95% CI
Glaucoma	Optic disc	Superficial retina	0.277	0.121	0.093–0.149	44	0.335	0.258–0.412
		Deep retina	0.278	0.117	0.090–0.143	42	0.323	0.248–0.398
		Choroid	0.432	0.125	0.097–0.153	29	0.347	0.268–0.425
	Macula	Superficial retina	0.245	0.120	0.092–0.148	49	0.332	0.254–0.410
		Deep retina	0.254	0.117	0.090–0.145	46	0.325	0.249–0.401
		Choroid	0.389	0.114	0.088–0.140	29	0.316	0.243–0.389
Control	Optic disc	Superficial retina	0.234	0.065	0.055–0.075	28	0.181	0.153–0.209
		Deep retina	0.241	0.065	0.055–0.075	27	0.181	0.153–0.209
		Choroid	0.347	0.068	0.058–0.079	20	0.189	0.160–0.219
	Macula	Superficial retina	0.226	0.111	0.094–0.128	49	0.307	0.259–0.355
		Deep retina	0.241	0.115	0.097–0.133	48	0.320	0.270–0.369
		Choroid	0.333	0.106	0.089–0.122	32	0.294	0.248–0.339

*Sw* Within-subject standard deviation; *CI* Confidence interval; *CV* Coefficient of variation; *CR*_*w*_ Coefficient of repeatability

## Discussion

OCTA has been increasingly studied as a modality for diagnosing and monitoring patients with glaucoma, but very few, if any, have discussed the differences between different OCTA machines in their ability to diagnose various ocular diseases. This study aims to report the vessel density findings in healthy versus glaucomatous eyes using 2 different OCTA machines available in our institution, namely AngioVue and Swept Source.

Current evidence shows that lower vessel densities have been found in glaucomatous eyes compared to normal eyes in peripapillary area, optic disc and macular area [8–13, 21–23]. Similarly, we have found that vessel density is significantly reduced in glaucomatous eyes compared to that of healthy eyes using AngioVue machine. Contrary to previous studies, vessel density in glaucomatous eyes appeared to be greater than healthy eyes for optic disc scans when Swept Source machine was used for imaging. However, these differences were generally not statistically significant. Given that same participants underwent OCTA imaging via both AngioVue and Swept Source, it is difficult to explain the stark difference in the intra-visit results between the two machines except for technical differences either in image acquisition or interpretation.

Many studies have shown high diagnostic accuracy of optical coherence angiography using vessel density, especially that of peripapillary area, to differentiate glaucomatous and healthy eyes density [10, 21, 22, 24–27]. Our study showed findings consistent with above for AngioVue, and the AUROC for discriminating glaucomatous from healthy eyes was better for AngioVue (mean ranging 0.761–0.990 for optic disc, 0.934–1.000 for macula) than Swept Source (mean ranging from 0.113–0.365 for optic disc, 0.525–0.644 for macula).

Such differences in findings between different OCTA machines have been reported before. In a study by Rebolleda et al. [28], a comparison of two OCTA devices, AngioVue and AngioPlex, was performed in pseudoexfoliation (PXF) glaucoma versus primary open-angle glaucoma (POAG) and healthy subjects. In their study, only AngioVue detected a significantly lower capillary density in PXF glaucoma compared to POAG at similar glaucoma damage, and AngioVue-derived vascular parameters showed higher diagnostic capacity to discriminate among groups compared to AngioPlex.

Differences in diagnostic ability between OCTA devices can be explained via several reasons. First, the boundaries of tissue segmentation (AngioVue being 4 segmented layers, Swept Source being 3 segmented layers) and the area evaluated (whole macular area evaluated by Swept Source was larger 6x6mm than that by AngioVue 3x3mm while optic disc area evaluated by both machines were similar 3x3mm) differ between the two machines. Secondly, each modality employs different technology to quantify the motion contrast and also has different approaches to minimise motion artefacts and achieve optimal image quality with high resolution. Another study comparing 4 different OCTA modules also showed that there was better inter-grader agreement for Optovue compared to Topcon, and this was attributed to the fact that the grader reliability seemed to be associated with better quality of images and evaluated features [15]. Such technical differences between different OCTA machines may contribute to differing vessel density results in same subjects.

Our study also focused on test-retest reliability to determine if results are repeatable intra-visit for each of the machines. Such repeatability and reproducibility of OCTA has been described previously. In general, earlier studies demonstrated better repeatability of OCTA, with CV staying below 7% over a range of parameters, including those of macula, optic disc and peripapillary region, and for different OCTA algorithms used, be it split-spectrum amplitude decorrelation angiography (SSADA) algorithm, OCT-based microangiography (OMAG) or OCTA ratio analysis (OCTARA) [23]. However, for our study, there was higher CV up to 49% for healthy and glaucomatous eyes using either AngioVue or Swept Source. This shows much higher variability than current available evidence. A possible explanation is that our study includes a larger sample size compared to previous studies, making it difficult to reproduce low CVs to suggest good repeatability of OCTA. Secondly, current published studies had higher excluded signal strength index (SSI), where images with a SSI < 60 [29], 50 [30], and 45 [28] were excluded from their study, whereas our study excluded images with SSI < 40, which may affect the accuracy of data. Thirdly, due to technical differences, conversion of parameters and measured values between different instrument systems is difficult. Vessel density was analysed using a novel in-house developed software in this study. Contrary to the findings from AngioVue in this study and recent studies, the mean vessel density from Swept Source was generally higher in glaucoma patients compared to healthy controls for optic disc scans. On the other hand, H Akil et al. [31] reported even eyes with mild POAG could be differentiated from pre-perimetric glaucoma eyes, which also could be differentiated from normal eyes using Swept Source OCT angiography. These inconsistencies and high CV raises the question whether the novel in-house software was suitable in the vessel density calculation of Swept Source OCT angiography images.

Furthermore, we also found a difference in repeatability between two machines, where AngioVue was superior to Swept Source in its reproducibility of results. This suggests that the difference in technical algorithm of each device may affect the repeatability and reproducibility of intra-visit images. In fact, such difference in repeatability may also account for the finding above, where poorer repeatability found in Swept Source could have led to contradicting vessel density results and poorer discriminatory ability of Swept Source OCTA.

The findings from our study provide clinical significance in showing that there can be differing findings using different OCTA machines in monitoring optic disc and macular perfusion. While previous studies have shown that OCTA is a useful tool for monitoring vascular perfusion in ocular diseases, caution should still be exercised when interpreting data in view of significant test-retest variability within each machine and also across different machines.

Our study is not without its limitations. There was statistically significant difference in the demographics of our control and glaucoma group, where patients in control group had younger age than those in glaucoma group. Age in itself is an independent risk factor for microvascular changes in the eye [32], and other age-related eye conditions such as cataract can develop in elderly subjects, as evidenced by greater number of patients with cataract in the glaucoma group, which can also affect angiographic result. Male to female ratio was higher in glaucomatous than healthy eyes, and gender differences have also been shown in ocular blood flow [33]. We tried to mitigate this by performing age- and gender-adjusted analysis of the data. Furthermore, direct comparison between the two OCTA machines are not possible as AngioVue segments into 4 different layers compared to Swept Source which segments into 3 layers instead. Thus, despite having used same in-house software to analyse the vessel density from the two different machines, direct comparison of the vessel density values across different layers, superficial to deep, is not possible due to the difference in segmentation of layers between the two machines. It is also possible that our in-house software may be biased toward one system and not the other, hence preferentially having better results with one than the other. Moreover, image acquisition and interpretation can be affected by poor quality scans. After elimination of these poor-quality images, some participants only had one good quality scan which meant that we had to eliminate these participants from test-retest reliability calculations. This results in further reduction in our sample size as well. Lastly, we have only compared between two machines out of the many OCTA machines that are currently available and we suggest further studies to be undertaken to compare different OCTA machines in the future.

## Conclusion

OCTA is a new modality that provides high-speed, non-invasive, depth-resolved imaging of retinal and choroidal vasculature, and it has a great potential to expand our understanding, diagnosis and management of various ocular diseases including glaucoma.

There are currently no guidelines as to which machine and which specific scan protocol are best for clinical diagnosis and monitoring of glaucoma. To the best of our knowledge, our study is one of the first few of its kind to compare the diagnostic and repeatability indices in glaucoma versus controls using different OCTA machines. AngioVue consistently detected lower vessel density values in glaucomatous eyes compared to healthy eyes, had high diagnostic capability in distinguishing glaucomatous from healthy eyes and had better test-retest reliability compared to Swept Source.

Prior to establishing OCTA as a new imaging modality for management of glaucoma, findings should be reproducible not just within the same OCTA machine but across different OCTA machines to allow standardization of results. We propose that further wide-scale longitudinal studies should be undertaken to evaluate if there is any significant difference between the various machines in diagnosing and monitoring ocular diseases.

## Data Availability

The datasets used and/or analysed during the current study are available from the corresponding author on reasonable request.

## Abbreviations

AUROC: Area under the receiver operating characteristic
CV: Coefficient of variation
OCTA: Optical coherence tomography angiography
OCTARA: Optical coherence tomography angiography ratio analysis
RPC: Radial peripapillary capillaries
SSADA: Split-spectrum amplitude-decorrelation angiography
Sw: Within-subject standard deviation
tSNE: T-distributed stochastic neighbor embedding
